# Factors Affecting Continuance Intention in Non-Face-to-Face Telemedicine Services: Trust Typology and Privacy Concern Perspectives

**DOI:** 10.3390/healthcare11030374

**Published:** 2023-01-28

**Authors:** Lin Zhu, Xinshu Jiang, Junwei Cao

**Affiliations:** 1 Department of Business Administration, Kyung Hee University, Seoul 02447, Republic of Korea; 2 School of Business, Yangzhou University, Yangzhou 225127, China

**Keywords:** telemedicine, expectation confirmation theory, trust typology theory, personal privacy concerns

## Abstract

As the COVID-19 pandemic progressed, the resulting demand for telemedicine services increased. This research empirically examines the role of trust, privacy concerns, and perceived usefulness in customer confirmation, satisfaction, and continuing intention in telemedicine. A typology of trust was employed to classify trust into three dimensions and explore the mediating role of the three dimensions of trust in the relationship between satisfaction, perceived usefulness, and continued intention. We also examined the moderating role of personal privacy concerns in the relationship between trust and continued intention. For this study, we developed a structural equation model based on expectation confirmation theory and analyzed 465 questionnaires from Chinese online users. The expectancy confirmation theory (ECT) was reaffirmed by empirical evidence. The results showed that the relationship between perceived usefulness and satisfaction with continued intention is moderated by the three dimensions of trust. Privacy concerns can negatively moderate the relationship between structural assurance-based trust and continued intention. This study also identified potential threats to telehealth market growth alongside new insights.

## 1. Introduction

COVID-19 represents a major social concern, with increasing numbers of people fearing infection when receiving offline healthcare services, complicating public health governance [1,2]. Telemedicine is increasingly seen as an effective tool to help alleviate this problem [3]. Along with the growing demand for digital healthcare services based on no-contact services, mobile, and easy-to-use individual medical devices and application-based services are on the rise [4,5]. The global telehealth services market was projected to grow significantly from $49.9 billion in 2019 to $459.8 billion in 2030 [6]. In China, telemedicine usage has essentially doubled since 2019, already reaching 47% usage by the end of 2021 [7]. These investments have led to the creation of Pingan Health, Hao Doctor, DingXiang Doctor, ChunYu Doctor, and many other famous applications of telemedicine services [1].

Telemedicine refers to healthcare services that use Internet technology, including connected devices such as computers and mobile phones and platform technologies such as websites and applications [8,9]. By integrating advanced technologies such as IoT, AI, and Big Data with medical technologies, the use of various IT digital devices can facilitate telemedicine services [10]. While researchers have increasingly focused on telemedicine [1,11,12,13,14], critical aspects such as privacy, data security, and trust remain unexplored [15,16,17,18].

The development of a climate of trust is considered to be an essential task in the field of telemedicine services [11]. Some previous studies have considered trust from two perspectives: confidence in the foreseeability of one’s expectations and confidence in the goodwill of others to maintain their commitments [19,20]. From an exchange perspective, trust can be built when one party believes in the exchange partner’s reliability and integrity; additionally, trust is complex and should be studied from a multidimensional perspective [21]. Recent literature typically views trust as a one-dimensional concept, failing to specify the unique impact of each type of trust on loyalty behavior [11,22]. It is unclear which types of trust [18,23,24] are most important for customer repurchase intention.

In addition to trust, important factors related to telemedicine processes in the context of IoT adoption include privacy concerns, security concerns, and regulatory issues [25,26]. IoT can be used to collect and process large amounts of telehealth data, but a lack of privacy and confidentiality may prevent users from sharing their data [27]. Concerns about online privacy have grown exponentially due to sweeping changes in the collection, storage, mining, and marketing of consumer data [28,29,30]. Therefore, the study of privacy concerns in relation to perceptions of data misuse is critical to an understanding of user behavior.

By analyzing these previous studies, we identified the following gaps: (1) There is no multidimensional perspective of trust available to explore why customers continue to use telemedicine; (2) There is no research on privacy issues related to perceived data misuse in telemedicine, which uses IoT as its technical context. To address this problem, in this study, we followed a modified expectation confirmation theory (ECT) [31,32] and employed the typology of trust developed in McKnight [33], which distinguishes between structural assurance-based trust, platform-based trust, and physician-based trust in telemedicine and can determine the relative impact of each category on the relationship of perceived usefulness, satisfaction, and continuance intention [34,35]. We also investigated the moderating effect of privacy issues on the relationship between trust and continuance intention.

The purpose of this study can be summarized as follows: First, to empirically validate the effectiveness of the expectation confirmation theory (ECT) in the process of telemedicine services. Second, to extend trust into three dimensions with the aim of elucidating how these dimensions affect behavioral intention to consistently use telemedicine services. Third, to investigate the moderating role of privacy protection during telemedicine services.

## 2. Theoretical Background and Hypothesis Development

### 2.1. Expectation Confirmation Theory

Expectation confirmation theory (ECT) is often used to identify the relationship between customer confirmation, satisfaction, and intention to continue in the marketing field [32]. However, Bhattacherjee extended ECT to the field of information management by arguing that whether users continue to use an information system is a decision similar to the decision-making behavior associated with consumer repurchasing and, therefore, proposed an information system continuity model [31]. ECT can be used to explore the intention to continue using information technology from a utility perspective such as perceived usefulness [31,36,37].

ECT suggests that a positive (negative) confirmation of a product’s performance in line with expectations leads to satisfaction (dissatisfaction), and thus, to the (lack of) intention to continue purchasing [31,32]. Previous research applying ECT has shown that user satisfaction is influenced by perceived usefulness and confirmation and that user satisfaction can lead to continuous intention [34,38]. If the expectation of a user to use a telemedicine service is confirmed and a perception of perceived usefulness is created, these factors are likely to satisfy the user and create an intention to continue to use the service. In other words, users will be satisfied and continue to use telemedicine services if they perceive usefulness in the performance of those services, which is in line with the existing literature. Therefore, the following hypotheses are proposed:

**H1a.**  *User confirmation has a positive impact on user-perceived usefulness.*

**H1b.**  *User confirmation has a positive impact on user satisfaction.*

**H2a.**  *User-perceived usefulness has a positive impact on user satisfaction.*

**H2b.**  *User-perceived usefulness has a positive impact on telemedicine continuance intention.*

**H3.**  *User satisfaction has a positive impact on telemedicine continuance intention.*

### 2.2. Typology of Trust

Trust is involved in every transaction on an Internet platform [39,40], and this factor is relevant to both platform-based trust and service-provider-based trust [41]. The multi-dimensional and multi-functional nature of trust is a very complex issue [42]. The antecedents of trust have been identified as knowledge-based, institutional (structural assurance and situational norms), calculative, cognitive (illusion of control), and personal [43]. Trust based on institutions refers to an individual’s overall perception of the transaction environment, including the institutional environment, in which there are structural assurances of trust on the Internet and regulatory environment, and the trust in specific online vendors in which there is platform-based trust [41,44,45]. Researchers have argued that the components of personality-based trust are largely dependent on the personal attitudes, trustworthiness, self-image, and influence of people in society [44,46]. Structural assurance-based trust applies to trust in institutional frameworks, including laws and regulations, rather than trust in the transaction itself [44,45]. Platform-based trust is the perception of a consumer towards a particular application or Internet-based platform in terms of the extent to which that technology is trustworthy, while personality-based trust is defined as trust in individuals, such as Airbnb hosts and DiDi drivers [41,44].

Consumer trust needs to be built both before and after the purchase experience [47,48]. Comparatively, pre-trust has a pivotal role in the initial engagement of the consumer [49,50], while post-trust, which is based on the consumer experience, has a more significant influence in repurchase intention [51,52]. Satisfaction based on a transaction increases trust in the structure and service of the provider’s platform [41]. For example, as the driver is the direct service provider during the car-riding process, passenger satisfaction affects the trust of the driver [44]. Thus, the satisfaction and perceived usefulness of telemedicine affect the trust of the structure, platform, and physician. Therefore, if users develop a perception of usefulness by using telemedicine services to their satisfaction, this perception may increase not only their trust in cybersecurity, but also their trust in the platform and the physician as the direct service provider. Therefore, the following hypotheses are proposed:

**H4a.**  *User-perceived usefulness has a positive impact on structural assurance-based user trust.*

**H4b.**  *User-perceived usefulness has a positive impact on platform-based user trust.*

**H4c.**  *User-perceived usefulness has a positive impact on physician-based user trust.*

**H5a.**  *User satisfaction has a positive impact on structural assurance-based user trust.*

**H5b.**  *User satisfaction has a positive impact on platform-based user trust.*

**H5c.**  *User satisfaction has a positive impact on physician-based user trust.*

To investigate the antecedents and impact of trust on (re)purchase intentions, numerous empirical studies have been conducted [41,44,53,54,55]. Repurchase intentions increase based on trust in structural guarantees and service provider platforms, which has also been shown to increase based on trust in Airbnb hosts and DiDi drivers [41,44]. Thus, in the telemedicine process, increased trust in structural assurance and the service provider platform increases continuance intention, as does increased trust in the physician. In other words, user confidence in structural guarantees can significantly alleviate user concerns about uncertainty in the online environment, as well as increase trust in online platforms and trust in physicians, all of which can positively impact user persistence. Therefore, the following hypotheses are proposed:

**H6a.**  *Structural assurance-based user trust has a positive impact on telemedicine continuance intention.*

**H6b.**  *Platform-based user trust has a positive impact on telemedicine continuance intention.*

**H6c.**  *Physician-based user trust has a positive impact on telemedicine continuance intention.*

### 2.3. Personal Privacy Concerns

Privacy concerns have been identified as an important antecedent of online behavior, with consumers expressing strong control and protection needs [56]. Using the Internet for transactions often requires the disclosure of large amounts of personal information, whether for business purposes (e.g., credit card data and shipping information) or e-commerce requirements [57]. Routine Internet activities have been shown to correlate with an increased incidence of Internet fraud, and falling victim to Internet fraud is associated with increased online privacy concerns [58].

By modeling privacy concerns as a precursor to trust, a negative correlation between trust and privacy concerns in online transactions has been demonstrated by numerous empirical studies [59,60,61,62]. Compared to the risk of actual financial losses due to losses of private data, the perceived risk of losing personal privacy itself is sufficient to negatively affect the intention to perform [63]. In addition, potential moderators of the negative effects of privacy concerns on behavioral intentions in the context of personalized online interactions have previously been examined [64]. When using telemedicine services in the context of Internet technology, users must trust in the structural guarantees and platforms to ensure the security of their private information and also trust that doctors will not disclose patient information. This means that users will be more likely to rely on structural assurance-based trust, platform-based trust, and physician-based trust to ensure privacy and security when using telemedicine in order to eliminate personal privacy concerns. Therefore, the following hypotheses are proposed:

**H7a.**  *The relationship between structural assurance-based user trust and telemedicine continuance intention is negatively moderated by personal privacy concerns.*

**H7b.**  *The relationship between platform-based user trust and telemedicine continuance intention is negatively moderated by personal privacy concerns.*

**H7c.**  *The relationship between physician-based user trust and telemedicine continuance intention is negatively moderated by personal privacy concerns.*

## 3. Research Model and Questionnaire Survey

### 3.1. Research Framework

**Figure 1:**
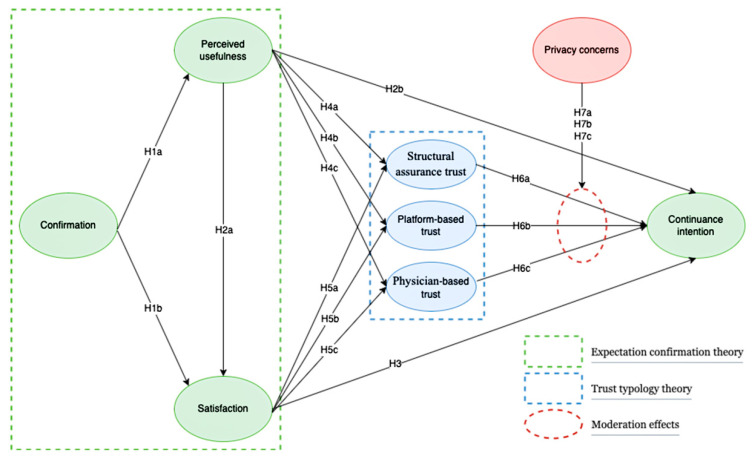
Research model.

In this study, we proposed a research framework (Figure 1) composed of eight variables. As shown in Figure 1 this study followed a modified expectancy confirmation theory (ECT) and employed a typology of trust, distinguishing between structural assurance-based trust, platform-based trust, and physician-based trust. For the process of using telemedicine, we actively identified whether the actual performance of telemedicine services meets expectations by confirming with users, in order to further identify user satisfaction and perceived usefulness of the platform and to determine the user’s intention to continue using such technology. This structural relationship can be influenced by three dimensions of post-trust: structural assurance-based trust, platform-based trust, and physician-based trust. This relationship can also be moderated by the user’s personal concerns about privacy issues.

### 3.2. Questionnaire Survey

In this study, the scales for all variables were designed based on scales validated by existing research, with some modifications to the items to contextualize telemedicine use by users. The scales used to measure the constructs of confirmation, perceived usefulness, satisfaction, and continuance intention were adapted from research related to expectation confirmation theory [31,65]. The scales used to measure the three types of trust constructs were adapted from research related to trust typology theory [44,45]. The scales used for the measurement of the construct of personal privacy concerns were adapted from past research on privacy concerns [66,67].

In this research, the target population was recent or former users of telemedicine services. As the target population was predominantly users of online platforms, an online survey was employed for this research [68]. The data were collected by the largest online market research company in China, SoJump [69,70], which has a large database of over 6.2 million registered members from different cities across the country and can collect data from a random sample of specific people on request.

The questionnaire consisted of two sections, the first comprising questions about the structure of the study and the second consisting of questions on information about the respondents. The questionnaire was first drafted in English and then translated into Chinese. Once the initial questionnaire design was completed, it was reviewed and revised by experts in the field to check its validity. A small pre-test was also conducted to refine the questionnaire before the final online product was administered by the survey company to a random sample of 506 users who had recently or previously used telemedicine services. It was estimated that the entire questionnaire would take approximately two to five minutes to complete, so we removed 41 invalid responses that took less than two minutes to complete, resulting in 465 valid responses. The general characteristics of the respondents are detailed in Table 1.

## 4. Methods

First, there are two main methods that can be utilized to estimate structural equation models: covariance-based structural equation modeling (CB-SEM) and partial least squares structural equation modeling (PLS-SEM). Additionally, there are a number of options available to determine the choice between PLS-SEM or CB-SEM. CB-SEM is used when the goal is theory testing, theory confirmation, a comparison of alternative theories, or the structural model has circular relationships; alternatively, previous studies have employed a global goodness-of-fit criterion [71,72]. In this study, covariance-based structural equation modeling (CB-SEM) and the corresponding software package Amos 26 were used to test the theoretical models and hypotheses.

Second, to test the mediation effect of the structural model, a bootstrap maximum likelihood method with 2000 bootstrap samples was performed in a bias-corrected confidence interval. In the absence of observed indicators, specific mediating effects of the structure were tested using phantom variables and setting bootstrap confidence intervals corrected for bias at 95% [73].

Moreover, to assess the moderation effect of personal privacy concerns on the relationship of trust and user continuance intention, we adopted Ping Jr.’s (1995) approach [74,75]. We computed means for all of the construct indicators in the study model, mean-centered them, and then computed the variance of each factor loading, error variance, and latent variable variance.

## 5. Results

### 5.1. Bias Test Results

Before starting the analysis of the structure, two possible bias phenomena (non-response bias and common method bias) were identified to avoid further affecting the validity in the survey results. This step was performed because the present research was based on a self-administered questionnaire, with both the dependent and independent variables derived from the same sample of respondents. First, to appraise non-response bias, we compared the measurements of early and late responders. The *t*-test results revealed non-significant differences between the groups, which indicates that a non-response bias was lacking in this research [76]. Second, common method bias is an easily apparent problem in questionnaires. Generally, when survey responses from a single medium (i.e., online) are collected, the survey responses may appear skewed or show a tendency to underestimate or exaggerate results in some way. To appraise common method bias, we applied Harman’s single-factor approach. The test results showed that the first factor represented 37.639% of the total variance. This result is below the recommended threshold of 40%, which indicates that there was no common method bias in this research [77,78].

### 5.2. Measurement Model Results

This study used structural equation modeling to analyze the data, starting with conducting confirmatory factor analysis (CFA) on the structural model to verify the overall fitness of the model and analyze the reliability and validity of the construct. Accordingly, the CFA results are presented in Table 2. The results of the CFA showed a satisfactory model fit, with *χ*^2^ = 425.402, df = 296, *p* < 0.001, *χ*^2^/df = 1.437 < 3, SRMR = 0.029 < 0.05, RMSEA = 0.031 < 0.05, CFI = 0.982 > 0.96, TLI = 0.979 > 0.96, and IFI = 0.982 > 0.95 [79,80].

As shown in Table 2, we used Cronbach’s alpha and composite reliability (CR) to identify reliability and thereby determine the internal consistency of the measurement model. Cronbach’s alpha values ranged from 0.765 to 0.895, while the CR values ranged from 0.771 to 0.897, exceeding the recommended value of 0.7 [79,81]. When testing for convergent validity (CV), the common rule is to check that the standard loadings for each item are 0.7 or higher. Additionally, the average variance extracted (AVE) must be higher than the recommended value of 0.5 [79]. Here, all factor loadings exceeded 0.7, and all AVE values exceeded 0.5.

In addition, research constructs should have acceptable discriminant validity (DV), which implies that each construct should represent the square root of AVE values greater than the correlation coefficient value of the constructs [81]. As shown in Table 3, the square root of the AVE values for each construct exceeded the corresponding correlation coefficient values between the constructs.

### 5.3. Structural Model Results

**Figure 2:**
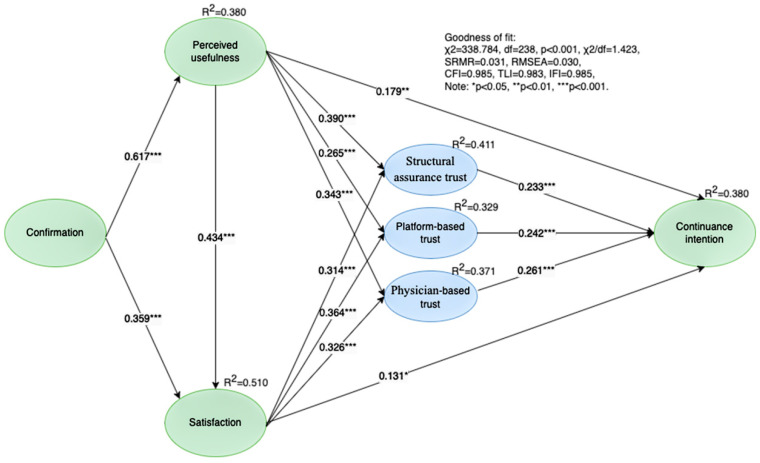
Results of the research model.

The structural model showed a satisfactory model fit with *χ*^2^ = 338.784, df = 238, *p* < 0.001, *χ*^2^/df = 1.423 < 3, SRMR = 0.031 < 0.05, RMSEA = 0.030 < 0.05, CFI = 0.985 > 0.96, TLI = 0.983 > 0.96, and IFI = 0.985 > 0.95, where the overall model fit indexes all met the recommended values [79,80]. The squared multiple correlations (R2) of the endogenous variables are greater than 0.3. As shown in Figure 2 and Table 4, in the complete structural model, all of the direct paths were significant.

Table 4 summarizes the direct and indirect effects. For the direct effects, the relationships between confirmation, perceived usefulness, and satisfaction were statistically significant, and the relationships between perceived usefulness, satisfaction, and continuance intention were also significant. Thus, H1a, H1b, H2a, H2b, and H3 are supported. The effects of perceived usefulness and satisfaction on the three trust components were statistically significant as well, thus supporting H4a–c and H5a–c. Statistical significance was also observed for the effects of structural assurance-based trust, platform-based trust, and physician-based trust on continuance intention, which supported H6a–c.

In terms of the indirect effects, the results showed the mediating effect of the three trust components on the relationship between perceived usefulness and continuance intention, as well as satisfaction and continuance intention, which were all statistically significant. Therefore, structural assurance-based trust, platform-based trust, and physician-based trust play a mediating role between perceived usefulness and continuance intention, and also between satisfaction and continuance intention.

Finally, in terms of moderation effects, among the relationships between the three trust components and continuance intention, only the interaction effects of structural assurance-based trust and privacy concerns were significant, as shown in Table 5. Thus, H7a was supported, while H7b and H7c were rejected.

## 6. Discussion and Implications

### 6.1. Key Findings

This study followed a modified expectancy confirmation theory (ECT) [31,32] to understand why users continue to use telemedicine services based on three dimensions of trust. The moderating influence of privacy concerns on the interrelationship between user trust and the intention to continue using was also explored. Based on the foundation of these elements, we derived the following key findings. First, we found empirical support for the applicability of the modified ECT to telemedicine services in China. Our results show that satisfaction and perceived usefulness of telemedicine services, when confirmed to meet user expectations, lead to an intention to continue using telemedicine services, which is fully consistent with our hypothesis.

Second, we also examined trust as one of the internal mechanisms of ECT [40]. When users are satisfied and find telemedicine services useful, a sense of trust in the institutional environment of the Internet (i.e., structural assurance) and in the Internet platform and service provider (i.e., the physician) is generated, which increases the user’s willingness to continue using telemedicine services. According to the typology of trust [33,45], three dimensions of trust (i.e., structural assurance-based trust, platform-based trust, and physician-based trust) were tested in this study.

Third, when examining the moderating effect of privacy concerns on the interrelation between user trust and intention to continue using, we found that privacy concerns have a negative moderating effect between user trust based on the structural guarantees of the Internet and intention to continue using but not on platform- and physician-based trust. This result can be explained by the fact that if users encounter a privacy violation and first consider the weak regulatory environment of the Chinese Internet market [82], there is a significant negative moderating effect between trust based on the structural guarantees of the Internet environment and intention to continue using when users perceive the privacy violation to be serious.

### 6.2. Theoretical Contributions

The theoretical contributions of this study include, first, new empirical evidence for the applicability of expectancy confirmation theory (ECT) to telemedicine services in the information domain [83], and an advancement of ECT theory by exploring the internal mechanisms linking the relationships between satisfaction, perceived usefulness, and continuance intention, which were able to accurately test the relationship between trust and ECT theory. Taking a multidimensional perspective on trust [33,45], the division of trust into three separate dimensions based on structural assurance-based trust, platform-based trust, and service-provider-based trust showed that all three trust dimensions have different degrees of mediating effects between users’ perceived usefulness and satisfaction and continued willingness, providing new evidence for understanding the different types of trust in the telehealth literature.

Second, we provided new insights into the role of trust in the context of telemedicine. As pre-trust purchase intentions have been extensively studied [84,85], we complemented these studies by shifting our focus to post-trust reuse intentions in telemedicine. Our study is also one of the few empirical tests to validate the interactive effects of post-trust types on user intentions to persist in telemedicine from all three dimensions. Thus, this study provides new evidence for understanding different types of post-trust in the sharing economy literature.

Finally, our findings are important because the literature on privacy issues in telemedicine in relation to Internet technologies is sparse [86,87], and our study provides new empirical evidence showing that users who experience privacy violations first consider the weak regulatory environment of the Chinese Internet market, rather than the Internet platform or service provider, thereby providing a useful starting point for future research.

### 6.3. Practical Contributions

Our findings suggest that user-perceived usefulness and satisfaction are critical to increasing user structural assurance-based trust, platform-based trust, and service-provider-based trust and further influence continued intention to use. User satisfaction has the greatest impact on platform-based trust, while user physician-based trust is the most important factor in continued intention to use telemedicine. Therefore, telehealth companies need to focus on increasing investments in building their platforms and fostering good physician-based trust while maintaining their infrastructure, e.g., by actively and publicly displaying physician research results and recent case outcomes. Platforms could also take a positive stance in responding to some negative user messages about physicians or the platform by explaining and responding to them, understanding the specific reasons for their negative messages, and making targeted improvements. By increasing the perceived usefulness of the platform and the satisfaction of the customer, trust in the platform and the doctor can be increased, which would, in turn, increase willingness to continue using the telemedicine service.

Second, we found that the persistent privacy problems on Internet platforms are still mainly due to the weak regulation of the Internet market. Therefore, Internet regulators need to develop measures to increase the confidence of users in the security of their privacy and the awareness of users by advocating for their protection. Telemedicine platform companies should design comprehensive privacy authorization systems that provide good privacy and security for users so that medical staff have sufficient access to patient information without revealing relatively confidential private information about the patient. Access to patient data, but not patient details (location, name, relevant age, gender, etc.) should be granted to doctors for the purpose of diagnosing a medical condition. In the long run, companies will benefit from adopting a user-centric approach to privacy that gives consumers sufficient control and choice, as these measures can increase user trust and loyalty.

Finally, the higher the perceived usefulness of users, the higher their satisfaction; in order to create a good medical experience, companies need to actively train and supervise doctors, including training in the use of relevant equipment [88,89,90], to improve medical services, and thus, the user experience through improved operations. The establishment of a supervisory department to regulate conflicts between doctors or the platform and users will help to resolve conflicts at the source and maximize the quality of the healthcare environment. Users also need to be actively informed about telemedicine services, and the information promoted by the company will increase awareness of telemedicine services and, ultimately, the willingness to use such services.

## 7. Conclusions

### 7.1. Limitations and Future Directions

The framework of the current study was limited in that it did not consider the influence of other antecedents or consequences on the type of trust and continuance intentions in the current context. Future research should include other elements of the trust typology. In particular, due to the nature of the data collected through the questionnaire, we were unable to examine both the antecedents and consequences of the trust typology in this study. Future research could use different data to examine both the antecedents and consequences of trust in both dimensions of trust. While the social network concept can measure the degree of trust between experts [91,92], the trust relationship between the telemedicine platform and the user influences the evaluation, and future research is required.

Previous research has shown that despite online privacy concerns and worries, online consumers sometimes deliberately disclose personal privacy information for some benefit, accept conditions such as being tracked, and do not take adequate privacy precautions [93]. Future research will include this privacy paradox in which users’ behavior runs counter to their privacy concerns.

Moreover, we only measured the intention of the user to continue using the service and did not examine the actual behavior of the user. Although there is no substantial discrepancy between intentions and actual behavior, intentions and actual behavior are not exactly equivalent. Future research could analyze the actual behavior of users by collecting data on their actual activities.

By looking at our research sample, it can be seen that the majority of the sample was young, and, for various reasons, we only conducted an online questionnaire using the largest research company in China. Therefore, our findings do not represent a comprehensive picture of all age groups using telemedicine. In the future, we could use different methods such as combining online and offline data to enrich the diversity of the sample.

### 7.2. Conclusions

This study employs an integrated research model based on expectancy confirmation theory (ECT), trust type theory, and privacy concerns to comprehend why users of telemedicine services continue to use them from a multifaceted perspective of trust. The expectation confirmation theory (ECT) is again empirically confirmed. According to the findings, telemedicine’s perceived usefulness and user satisfaction are positively correlated. The findings imply that users will continue to use the service if they have confidence in the structural assurance, the platform, or the physician, and that this trust is driven by perceived usefulness, satisfaction, and confirmation. In addition, we discovered that the three dimensions of trust act as a moderator in the relationship between perceived usefulness and continued intention satisfaction. Finally, we explored that trust based on structural assurance plays a more positive role in continued intention when users perceive privacy concerns. These findings offer fresh perspectives and insights into potential dangers to the telemedicine market’s expansion.

## Figures and Tables

**Table 1:** General characteristics of the respondents.

		*N*	%
Gender	Male	207	44.52%
	Female	258	55.48%
Age	18–25 years	183	39.35%
	26–35 years	178	38.28%
	36–45 years	103	22.15%
	≥46 years	1	0.22%
Monthly income	<3000 RMB	24	5.16%
	3000–5000 RMB	52	11.18%
	5001–8000 RMB	166	35.70%
	8001–10,000 RMB	138	29.68%
	>10,000 RMB	85	18.28%
Education	Less than high school	34	7.31%
	High school	143	30.75%
	4-year college	268	57.63%
	Graduate student or higher	20	4.30%
Occupation	Students	20	4.30%
	Company employees	272	58.49%
	Civil servants	56	12.04%
	Self-employment	64	13.76%
	Others	53	11.40%
Total		465	100.00%

**Table 2:** Measurement model results.

Constructs	Items	Loadings	Cronbach’s α	CR	AVE
Confirmation	1. My experience with using telemedicine was better than I expected.	0.821 #	0.887	0.887	0.663
	2. The level of service provided by telemedicine was better than I expected.	0.803 ***			
	3. My expectations for telemedicine services were correct.	0.831 ***			
	4. Overall, most of my expectations for using telemedicine were confirmed.	0.801 ***			
Perceived usefulness	1. I think the telemedicine mobile application is very useful.	0.835 #	0.895	0.897	0.686
	2. I think using telemedicine could help me improve my health.	0.859 ***			
	3. I think the service offered by telemedicine is very useful.	0.823 ***			
	4. On the whole, I think it’s useful to use telemedicine.	0.793 ***			
Satisfaction	1. Your thoughts about telemedicine services: Very displeased/Very pleased.	0.777 #	0.886	0.889	0.667
	2. Your thoughts about telemedicine services: Very dissatisfied/Very satisfied.	0.833 ***			
	3. Your thoughts about telemedicine services: Very frustrated/Very contented.	0.845 ***			
	4. You think telemedicine services are: Absolutely terrible/Absolutely delightful.	0.810 ***			
Structural assurance-based trust	1. The Internet has sufficient security measures to allow me to use it for personal matters.	0.872 #	0.839	0.844	0.644
	2. I am sure the legal and technical structures protect me from problems on the Internet.	0.833 ***			
	3. I believe that encryption and other technical advances on the Internet will allow me to do business there more safely.	0.821 ***			
Platform-based trust	1. Telemedicine applications meet my needs as a consumer.	0.843 #	0.844	0.849	0.652
	2. Telehealth applications could provide me with good health services.	0.768 ***			
	3. The services provided by telemedicine applications are reliable.	0.796 ***			
Physician-based trust	1. The telemedicine doctors are very professional.	0.774 #	0.765	0.771	0.530
	2. The telemedicine doctors are honest.	0.794 ***			
	3. The telemedicine doctors have been very helpful to me.	0.852 ***			
Continuance intention	1. I would continue to use telemedicine.	0.729 #	0.873	0.880	0.709
	2. I would be using telemedicine as often as I do now.	0.657 ***			
	3. I would increase the frequency with which I use telemedicine in the future.	0.792 ***			
Personal privacy concerns	1. I feel concerned that the information I have submitted online may be misused.	0.739 #	0.821	0.836	0.629
	2. I feel worried that someone would be able to find information about me on the Internet.	0.832 ***			
	3. I feel concerned that information submitted online will be used in unintended ways.	0.806 ***			

Model fit indexes: *χ*^2^ = 425.402, df = 296, *p* < 0.001, *χ*^2^/df = 1.437, SRMR = 0.029, RMSEA = 0.031, CFI = 0.982, TLI = 0.979, and IFI = 0.982. Note: *** *p* < 0.001; # factor loading was fixed at 1, so that the *p*-value is not presented.

**Table 3:** Correlation and square root of the AVE table.

	1	2	3	4	5	6	7	8
1. Confirmation	0.814							
2. Perceived usefulness	0.616	0.828						
3. Satisfaction	0.627	0.656	0.817					
4. Structural assurance-based trust	0.423	0.593	0.567	0.803				
5. Platform-based trust	0.363	0.505	0.540	0.357	0.807			
6. Physician-based trust	0.412	0.553	0.548	0.476	0.361	0.728		
7. Continuance intention	0.538	0.665	0.649	0.617	0.578	0.625	0.842	
8. Personal privacy concerns	0.048	−0.153	−0.064	−0.177	−0.136	−0.171	−0.208	0.793

Note: The square root of AVE for each research construct is presented on the diagonal. Correlations between constructs are under the square root of AVE.

**Table 4:** Results of hypothesis testing and mediation effects.

Hypothesis		β	*t*-Value	Results
H1a	Confirmation → Perceived usefulness	0.617 ***	12.166	Supported
H1b	Confirmation → Satisfaction	0.359 ***	6.386	Supported
H2a	Perceived usefulness → Satisfaction	0.434 ***	7.613	Supported
H2b	Perceived usefulness → Continuance intention	0.179 **	2.976	Supported
H3	Satisfaction → Continuance intention	0.131 *	2.168	Supported
H4a	Perceived usefulness → Structural assurance-based trust	0.390 ***	6.067	Supported
H4b	Perceived usefulness → Platform-based trust	0.265 ***	4.007	Supported
H4c	Perceived usefulness → Physician-based trust	0.343 ***	4.904	Supported
H5a	Satisfaction → Structural assurance-based trust	0.314 ***	4.895	Supported
H5b	Satisfaction → Platform-based trust	0.364 ***	5.350	Supported
H5c	Satisfaction → Physician-based trust	0.326 ***	4.637	Supported
H6a	Structural assurance-based trust → Continuance intention	0.233 ***	4.486	Supported
H6b	Platform-based trust → Continuance intention	0.242 ***	5.042	Supported
H6c	Physician-based trust → Continuance intention	0.261 ***	4.848	Supported
Mediation Effect of Trust				Indirect
	Perceived usefulness → Structural assurance-based trust → Continuance intention			0.048 ***
	Perceived usefulness → Platform-based trust → Continuance intention			0.034 ***
	Perceived usefulness → Physician-based trust → Continuance intention			0.047 ***
	Satisfaction → Structural assurance-based trust → Continuance intention			0.039 ***
	Satisfaction → Platform-based trust → Continuance intention			0.047 ***
	Satisfaction → Physician-based trust → Continuance intention			0.035 ***

Model fit indexes: *χ*^2^ = 338.784, df = 238, *p* < 0.001, *χ*^2^/df = 1.423, SRMR = 0.031, RMSEA = 0.030, CFI = 0.985, TLI = 0.983, and IFI = 0.985. Note: * *p* < 0.05, ** *p* < 0.01, and *** *p* < 0.001.

**Table 5:** Results of the moderation effects.

	Relationships	β	*t*-Value
H7a	Structural assurance-based trust × privacy concerns → Continuance intention	−0.084 *	2.161
H7b	Platform-based trust × privacy concerns → Continuance intention	−0.048	1.133
H7c	Physician-based trust × privacy concerns → Continuance intention	−0.049	0.876

Model fit indexes: *χ*^2^ = 489.355, df = 127, *p* < 0.001, *χ*^2^/df = 3.853, SRMR = 0.095, RMSEA = 0.078, CFI = 0.900, TLI = 0.880, and IFI = 0.901. Note: * *p* < 0.05.

## Data Availability

The data presented in this study are available upon request from the corresponding author. The data are not publicly available for ethical reasons.
